# Association between bat-predator species richness and Nipah virus spillover risk in Bangladesh

**DOI:** 10.1016/j.onehlt.2025.101274

**Published:** 2025-11-15

**Authors:** Jun-Sik Lim, Kyung-Duk Min

**Affiliations:** a Saw Swee Hock School of Public Health, National University of Singapore, Singapore; b Centre for Nature-based Climate Solutions, National University of Singapore, Singapore; c College of Veterinary Medicine, Chungbuk National University, Cheongju, Republic of Korea

**Keywords:** Biodiversity, Predator, Spillover, Nipah virus

## Abstract

Species biodiversity is considered to reduce infectious diseases spillover from wildlife to human. However, despite the potential role of predator biodiversity in this process through trophic cascade, few studies have addressed this issue. In this study, we investigated the association between predator biodiversity and spillover risk, using Nipah virus infection in Bangladesh as an example, where spillover from bats to human has been reported since 2021.

We defined counties of Bangladesh as epidemiological units. From three Orders (Strigiformes, Accipitriformes, and Falconiformes) known as bat-preying predators, we extracted 39 species occurrences data and then built species distribution model using MaxEnt algorithm with climate and environmental predictors, also incorporating a bias grid to account for reporting bias. Species presence and richness were estimated under varying classification thresholds and species subsets reported to prey bats to allow sensitivity analyses, yielding 12 measures of species richness for each Order. We then used spatial model to identify the association between the species richness and the counties with spillover event, while adjusting for confounders.

Results showed that greater biodiversity of owls (Strigiformes) is likely to reduce the risk of Nipah virus spillover. In contrast, the biodiversity of eagles (Accipitriformes) and falcons (Falconiformes) have a potential of positive association, but evidence was insufficient. This result can be explained by the differences in activity rhythms. Owls share a nocturnal activity rhythm with bats, providing more opportunities to prey on bats and reduce their activity, thereby lowering spillover risk. In contrast, eagles and falcons are diurnal, and thus less likely to suppress bat activity directly. Instead, they may suppress species that compete with bats for food, inadvertently facilitating bat activity and increasing spillover risk. These results suggest that biodiversity should be more explicitly considered in public health governance and spillover prevention strategies.

## 1 Introduction

Species biodiversity shapes the emergence of zoonotic infectious diseases. As biodiversity enriches, the interaction between natural host of a zoonotic pathogens and their vectors reduces. Simultaneously, natural hosts suffer from competing with more competent hosts. Those relieve the risk of diseases within natural host populations and thus lower the spillover event [1]. Several studies uncovered this phenomenon, called as dilution effect, particularly in parasitic diseases [2] and a few viral infectious diseases such as Lassa fever [3].

Predator biodiversity can serve as a potential determinant for pathogen spillover to humans, as suggested by the distinct ecosystem characteristics known as trophic cascade [[4], [5], [6]]. The increased predator biodiversity makes the prey less populated [5], and inactive [4], thus reliving spillover risk to humans [6]. Despite this the potential association, only a limited studies explored and identified the protective effect of predators on zoonotic spillover events, mainly from rodent species. [7,8].

Nipah virus (NiV), which is one of the emerging zoonotic diseases in Bangladesh, establish ideal conditions to estimate the relationship between spillover and predator biodiversity. NiV has bats as its major reservoir [9] and can infect pigs and human, inducing respiratory and neurological symptoms with a high case-fatality rate ranging from 40 to 70 %. Since 2001, Bangladesh has reported the spillover of Nipah virus to human occurs annually [10] thorough palm sap contaminated by bat [11], showing the human-wildlife interaction.

The strengths of using NiV to study the relationship between spillover and predator biodiversity are as follows: (1) Bats serve as major reservoir for NiV and are widely distributed throughout Bangladesh [12]. The transmission of NiV in bat populations appears stable across the country [13]; (2) The primary predators of bats are owls (Order Strigiformes) followed by eagles (Order Accipitriformes) and raptor (Order Falconiformes) [9]. Their biodiversity can be estimated by building species distribution models (SDM) [[14], [15], [16]] using presence data retrieved from Global Biodiversity Information Facility (GBIF); (3) Surveillance for NiV has been conducted for whole country. Thanks to definite transmission routes and easily recognizable symptoms in human (palm sap consumption or direct contact with wildlife), (4) case due to spillover can be readily identified [17]. This context of NiV in Bangladesh provides a valuable opportunity to enhance our understanding on predator biodiversity and zoonotic spillover.

Thus, in this study, we aimed to identify the effect of the biodiversity of bat-preying species on the Nipah spillover risk, including Order Strigiformes, Accipitriformes, and Falconiformes within the context of Bangladesh.

## 2 Methods

### 2.1 Study design and dataset

This cross-sectional study defined a county of Bangladesh as an epidemiological unit. Outcome of this study is defined as counties whether the spillover event occurred or not during study period (2009 to 2018). We defined exposure variables of interest as county-level species richness for Order of Strigiformes*,* Accipitriformes*,* and Falconiformes, which were calculated from the species distribution models (SDM) developed in this study.

**Fig. 1:**
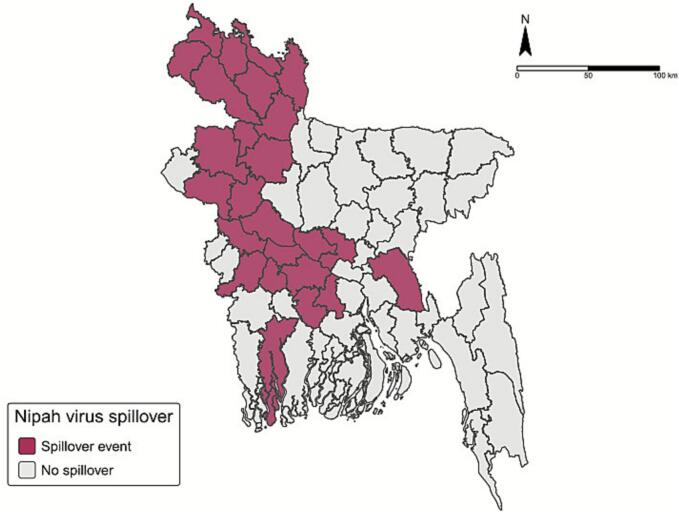
Spatial distribution of spillover event occurrences in Bangladesh during 2009–2018.

From previous published paper [18], we extracted county names that reported NiV spillover during 2009–2018. Among total 64 counties in Bangladesh, 24 counties reported NiV-positive patients suspected to be due to spillover during the study period (Fig. 1).

To estimate species richness, we built SDM using the species observation records extracted from GBIF [19]. We obtained total 65 species with 21,253 observations recorded in entire region of Bangladesh from GBIF, consisting of 19, 39, and 7 species in Order Strigiformes and Accipitriformes, and Falconiformes*,* respectively. We restricted our analysis into the 39 species of which observations data have complete geographical locations with over 30 observation records to support the performance of the developed SDMs. From these datasets, we additionally formulated dataset comprising bat-preying species to calculate the species richness of bat-preying species, following the information extracted from “*Birds of the World*” [20].

### 2.2 SDMs and species richness

To calculate species richness as county level, we constructed SDM for each species of each Family using the extracted dataset from the GBIF and make use of its prediction on the whole area of Bangladesh. We applied MaxEnt algorithm [21], which is commonly used in biodiversity research. MaxEnt uses presence-background data to estimate the habitat suitability of species in study region. In this study, presence data is observation records for each species and background data was selected randomly as a default setting of MaxEnt. Features were selected automatically. To build the best performance model, we included a total of 19 variables from *worldclim* data [22], and elevation [23]. We eliminated the least contributing variables using backward selection excluding the variables with less than 10 % of permutation importance. This exclusion was iterated until there was no variable with less than 10 % of the permutation importance. In each iteration for backward selection, we applied 10 folds cross-validation. With the final models, we modified averaged predictions from the 10 cross-validated final models as binary to show the predicted presence of the species by the threshold value from ROC curve to show the best performance as binary classifier.

To calculate the species richness as county level, if the predicted presence area of a species covered over a 50 % of area of county, we assigned presence of a species in a county. We applied this approach for all species of each Order. The predicted species presence maps as county level were generated. Then, we summed up these predicted maps for all species for Order Strigiformes, Accipitriformes, and Falconiformes, respectively.

As a sensitivity analysis for calculating the species richness, we applied additional approaches: (1) we restricted the species richness variable into the species reported that they prey bats (All species and bat-preying species); (2) due to the potential reporting bias that could be derived from the dataset, we built SDMs adjusted with the reporting bias using bias grid as background data representing the probability of observation using the human population density (random background, and bias grid background) [24]; (3) we also applied three different kinds of threshold (25 %, and 75 % area of county plus 50 %) for the criteria of assignment of presence of a species in a county to account for the potential misclassification bias. With these 12 types of conditions, totally, we calculated 12 different types of species richness variables for each Order.

### 2.3 Confounders

We included anthropogenic and climo-environment to adjust the confounding on the effect of species richness on NiV spillover risk (Table 1): human population density [25]; Gross domestic product *per capita* (GDP) [26]; Elevation [23]; Normalized Difference Vegetation Index (NDVI) [27]; Deforestation [28]. We also included Temperature of coldest quarter [22] and, precipitation of coldest quarter [22] following the previous study showing that low temperature and low precipitation in winter season were risk factors for NiV spillover [29]. We modified human population density, GDP, temperature of coldest quarter, and, precipitation of coldest quarter by averaging the values as county level. For NDVI, we extracted the yearly layers from 2010 to 2017 for whole Bangladesh and averaged these eight layers into one layer. Then, we calculated the averaged value of this layer for each county. Deforestation was extracted for 2010–2017 and made as binary as this value has little variation.

**Table 1:** Summary statistics of species richness variables.

Variables (unit)	Species	Background	Criteria for richness	Mean (min-max)	
				Counties with Nipah cases (*N* = 25)	Counties without Nipah cases (*N* = 39)
*Strigiformes*	All	Random	75 %	1.24 (0–3)	1.23 (0–4)
			50 %	2.12 (0–6)	2.05 (0–6)
			25 %	2.68 (0–6)	3.18 (0–7)
		Biased	75 %	0.92 (0–3)	1.51 (0–6)
			50 %	1.68 (0–6)	2.18 (0–6)
			25 %	2.52 (0–6)	3.15 (0–7)
	Bat-preying	Random	75 %	0.56 (0–2)	0.26 (0–2)
			50 %	1.04 (0–4)	0.74 (0–3)
			25 %	1.36 (0–4)	1.49 (0–4)
		Biased	75 %	0.08 (0–1)	0.33 (0–2)
			50 %	0.60 (0–4)	0.67 (0–2)
			25 %	1.24 (0–4)	1.26 (0–3)
*Accipitriformes*	All	Random	75 %	5.92 (2–14)	6.61 (2−13)
			50 %	7.76 (2–17)	8.74 (3–18)
			25 %	9.52 (2–18)	11.05 (5–21)
		Biased	75 %	5.40 (1–14)	6.13 (2−12)
			50 %	7.68 (2–15)	8.51 (2−20)
			25 %	9.32 (2–16)	11.18 (3−21)
	Bat-preying	Random	75 %	0.88 (0–3)	0.56 (0–3)
			50 %	1.36 (0–3)	0.92 (0–3)
			25 %	1.56 (0–3)	1.39 (0–3)
		Biased	75 %	0.76 (0–3)	0.59 (0–3)
			50 %	1.12 (0–3)	0.87 (0–3)
			25 %	1.36 (0–3)	1.41 (0–3)
*Falconiformes*	All	Random	75 %	0.48 (0–4)	0.31 (0–3)
			50 %	0.72 (0–5)	0.67 (0–4)
			25 %	1.24 (0–5)	1.41 (0–5)
		Biased	75 %	0.28 (0–3)	0.59 (0–4)
			50 %	0.56 (0–4)	1.15 (0–4)
			25 %	1.12 (0–4)	1.49 (0–5)
	Bat-preying	Random	75 %	0.4 (0–3)	0.21 (0–2)
			50 %	0.6 (0–4)	0.49 (0–4)
			25 %	0.96 (0–4)	1.03 (0–4)
		Biased	75 %	0.28 (0–3)	0.51 (0–3)
			50 %	0.56 (0–4)	1 (0–4)
			25 %	1 (0–4)	1.26 (0–4)

### 2.4 Estimating the association

We fitted logistic regression using species' richness for Strigiformes, Accipitriformes, and Falconiformes as main exposure variables with confounders. To test the spatial residual dependence of the models, Moran's I was applied. We assumed the spatial adjacency structure as the first-order contiguity between the counties. If it was statistically significant, Bayesian spatial conditional autoregressive logistic regression model was applied to account for residual spatial dependence [30]. As the species richness variables have 12 different types calculated from 12 conditions, totally 12 logistic regressions were built. Thus, we got 12 different types of odds ratio for each Order.

Prior distribution for variance of spatial random effect was defined as inverse gamma distribution with (alpha, beta). Prior for non-spatial random effect was as inverse gamma distribution with (alpha, beta). Prior distribution for the coefficients of all variables in the model were defined as normal distribution with mean of 0 and variance of 10.

All analysis were conducted with Bayesian Markov Chain Monte Carlo (MCMC) simulation using *R2OpenBUGS* package [31] linking R software to *OpenBUGS* [32]. Four chains of each posterior distribution were iterated with 50,000 times with 10,000 burn-in iterations. The posterior distribution of the chains were assessed to be converged by visually using trace plot and applying Gelman–Rubin–Brooks diagnostic [33]. The coefficients in the model were summarized by their median and 95 % credible intervals. The odds ratio whose 95 % credible interval did not overlap one was identified as statistically significant. Due to computational burden of MCMC iterations, parallel computing was performed using “*parallel*” and “*dclone*” packages. Data management, statistical analyses, and building SDM were conducted *SeegSDM*, and *dismo* packages [34,35] in R software.

## 3 Results

### 3.1 Species richness

A total of 78 SDM models were built, and each was used to project habitat suitability for 39 species with two background data assumptions. The area of under curve of ROC curve of those models showed 0.84 on average, ranging from 0.60 to 0.99.

Table 1 shows 12 different types of the calculated species richness from the built SDMs for each species, derived from two type of species data (all and bat-preying species) applying two distinct background data assumptions (random background and bias grid) along with three different thresholds to define the presence of species at county level (25 %, 50 %, and 75 % threshold).

### 3.2 Association

All of 12 logistic regression models in our analysis have a significant residual spatial dependence, as evidenced by statistically significant results from the Moran's I test. We used Bayesian spatial conditional autoregressive logistic regression model and confirmed that all of the posterior distributions of four MCMC from 12 models became well-mixed after 10,000 burn-in iterations and showed convergence after 50,000 iterations. Their Gelman–Rubin–Brooks diagnostic showed values less than 1.01, indicating good convergence.

**Fig. 2:**
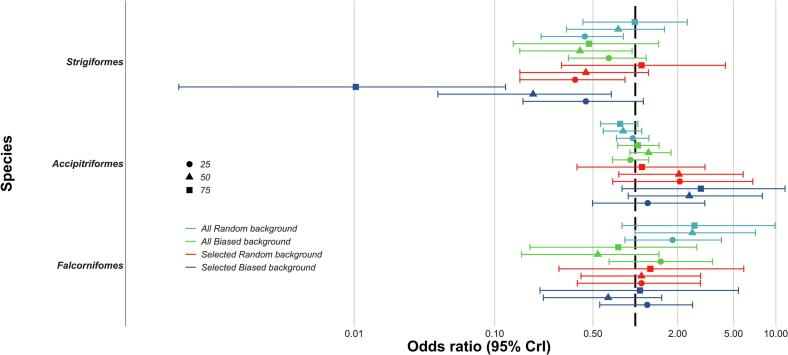
Estimates and 95 % credible intervals of odds ratios for the presences of *Strigiformes*, *Accipitriformes*, and *Falconiformes* under different species data and background data assumptions. Three thresholds were used to define species presence. Colors indicate the type of species data and background assumption, bars represent 95 % credible intervals, and shapes denote median values for each threshold.

Fig. 2 shows that the species richness of Family Strigiformes have a consistent and statistically significant negative association with the spillover risk of Nipah virus across models (Supplementary material A): Among 12 different assumptions, the estimated odd ratios (ORs) from 11 assumptions showed negative associations, out of which five estimates were statistically significant. Under the assumption of bat-preying biased-background model with a 75 % threshold, the odds ratio (OR) showed the strongest and significant negative association (median OR: 0.11, 95 % CI: 0.00–0.01). The only assumption of bat-preying random-background model with a 75 % threshold showed a weak positive association but lacking statistical significance (median OR: 1.1, 95 % CI: 0.30–4.37). The estimated odds ratios (ORs) showed weaker negative associations in all-species random-background models, with only the ORs from a 25 % threshold significant. The all-species biased background models estimated a stronger negative association compared to the ORs from all-species random-background models, but only the OR from a 50 % threshold was significant. Similarly, the association in bat-preying random background models to calculate species richness was estimated to be stronger toward negative association than in all-species random-background models. However, only the OR from a 25 % threshold was significant. The bat-preying biased-background models estimated the strongest ORs as 0.01 (95 % CI: 0.00–0.09) in a 75 % threshold.

Conversely, our results showed Accipitriformes, and Falconiformes richness have variable and no statistically significant associations with the spillover risk of Nipah virus (Fig. 2, Supplementary material A). For both Family, depending on the assumptions, either positive and negative association was found and half of them are close to null association, showing that assumption is likely to affects the trend of association.

## 4 Discussion

In this study, we investigated the association between bat predator biodiversity and Nipah virus spillover within the context of Bangladesh, exploring the diverse scenarios encompassing 12 different types of calculated species richness. Our findings suggest that higher species richness of *Strigiformes* is likely to reduce the risk of Nipah spillover. Conversely, the association between the species richness of Order *Accipitriformes* and *Falconiformes,* and the risk of Nipah spillover is less clear and requires further research.

The protective effect of Strigiformes biodiversity on Nipah spillover may be explained by their role as major predators of bats. As nocturnal species, they share the same active hours with bats, allowing them to exert strong predation pressure. This predation likely reduces overall bat activity and consequently lowers the risk of human–bat interactions. Other research suggested deforestation as risk factor for spillover events from bats to humans [36,37]. They proposed several hypothetical pathways in which deforestation can increase the spillover event, such as increased contact with crops due to the loss of their roosting sites. As deforestation and thus fragmented forest directly reduces available habitats, food, and water for wildlife, ultimately inducing biodiversity loss [38], our study is consistent with those previous studies by suggesting one of the pathways through which deforestation increase the risk of the spillover.

We found no statistically significant association between Accipitriformes, and Falconiformes on Nipah virus spillover to human. It does not correspond to the current existing knowledge that generalist predators, like Accipitriformes and Falconiformes, affect more prey biodiversity than specialist predators do [39,40]. This might be because, despite being generalists, Accipitriformes and Falconiformes are not major bat-preying species. Moreover, their diurnal hunting activity further limits their direct impact on bat populations, which are largely nocturnal. These hypotheses are speculative and require further empirical validation.

Human–animal interfaces play a crucial role in the dynamics of zoonotic transmission. The food web, as one of the distinct characteristics of an ecosystem, shapes these interfaces through prey–predator relationships. The present study provides empirical evidence supporting the role of predator biodiversity in influencing prey activity and, consequently, the risk of zoonotic spillover. Therefore, biodiversity conservation should be integrated into public health policy within the One Health framework.

Our study has several limitations. First, we assessed only the spatial variation in biodiversity and did not account for temporal dynamics, which may also influence spillover risk. Second, we excluded a subset of species within each order due to insufficient occurrence records. Because these species are likely rare, we believe their exclusion introduced only negligible bias into our results. A systematic investigation of predator biodiversity, combined with individual-level human spillover data rather than county-level data, would bring us closer to understanding the true association between biodiversity and Nipah virus spillover risk.

In this study, we found evidence that predator biodiversity, particularly that of bat-preying species, may exert a protective effect against the spillover of Nipah virus. These findings highlight the importance of biodiversity conservation not only for ecosystem stability but also for mitigating zoonotic disease risks. We suggest that biodiversity should be more explicitly considered in public health governance and spillover prevention strategies.

## CRediT authorship contribution statement

**Jun-Sik Lim:** Writing – review & editing, Writing – original draft, Methodology, Investigation, Formal analysis, Data curation, Conceptualization. **Kyung-Duk Min:** Writing – review & editing, Supervision, Methodology, Funding acquisition, Conceptualization.

## Declaration of competing interest

The authors declare that they have no known competing financial interests that could have appeared to influence the work reported in this paper.

## Data Availability

The datasets used in this study are available upon reasonable request.

## Appendix A Supplementary data

Supplementary material: Estimated adjusted Odds Ratios of each species for each scenario on Nipah spillover risk and DIC of the fitted spatial model. Image 1
