# Supplementary material for: Association between bat-predator species richness and Nipah virus spillover risk in Bangladesh
Source: One Health. 2025 Nov 15;21:101274. doi: 10.1016/j.onehlt.2025.101274 (PMC12666050; doi:10.1016/j.onehlt.2025.101274)
Supplement: Supplementary file 1 — Supplementary material: Estimated adjusted Odds Ratios of each species for each scenario on Nipah spillover risk and DIC of the fitted spatial model. [file mmc1.docx]

| **Variables** | **Background** | **Criteria for richness** | **Strigiformes**  **Odds Ratio**  **(95% CI)** | **Accipitriformes**  **Odds Ratio**  **(95% CI)** | **Falconiformes**  **Odds Ratio**  **(95% CI)** | **DIC** |
| --- | --- | --- | --- | --- | --- | --- |
| All | Random | 75% | 0.99  (0.42 – 2.35) | 0.78  (0.75 – 1.48) | 2.64  (0.80 – 9.87) | 80.99 |
|  |  | 50% | 0.76  (0.32 – 1.61) | 0.82  (0.59 – 1.11) | 2.55  (1.00 – 7.15) | 80.06 |
|  |  | 25% | 0.44  (0.21 – 0.82) | 0.96  (0.73 – 1.25) | 1.84  (0.84 – 4.10) | 76.36 |
|  | Biased | 75% | 0.47  (0.14 – 1.47) | 1.04  (0.75 – 1.48) | 0.76  (0.18 – 2.74) | 81.26 |
|  |  | 50% | 0.4  (0.15 – 0.95) | 1.24  (0.92 – 1.80) | 0.54  (0.16 – 1.47) | 78.75 |
|  |  | 25% | 0.65  (0.33 – 1.20) | 0.92  (0.69 – 1.25) | 1.52  (0.65 – 3.54) | 81.40 |
| Bat-preying | Random | 75% | 1.11  (0.30 – 4.37) | 1.11  (0.38 – 3.13) | 1.28  (0.29 – 5.91) | 85.29 |
|  |  | 50% | 0.45  (0.15 – 1.24) | 2.04  (0.76 – 5.85) | 1.11  (0.41 – 2.92) | 81.13 |
|  |  | 25% | 0.37  (0.15 – 0.84) | 2.07  (0.69 – 6.86) | 1.11  (0.39 – 2.90) | 76.55 |
|  | Biased | 75% | 0.01  (0.00 – 0.12) | 2.93  (0.81 – 11.62) | 1.08  (0.21 – 5.41) | 65.26 |
|  |  | 50% | 0.19  (0.04 – 0.68) | 2.42  (0.89 – 8.00) | 0.64  (0.29 – 5.91) | 74.48 |
|  |  | 25% | 0.45  (0.16 – 1.14) | 1.23  (0.50 – 3.12) | 1.22  (0.56 – 2.56) | 81.54 |

**Supplementary material 1. Estimated adjusted Odds Ratios of each species for each scenario on Nipah spillover risk and DIC of the fitted spatial model.**
